# Hybrid Mentorship of Medical Laboratories to Achieve ISO 15189:2012 Accreditation in Malawi: The University of Maryland Malawi Experience

**DOI:** 10.9745/GHSP-D-24-00254

**Published:** 2024-12-20

**Authors:** Hannaniah Moyo, Sophia Osawe, Charles Nyangulu, Philemon Ndhlovu, Visopo Harawa, Oscar Divala, Malango Msukwa, Talishiea Croxton, Natalia Blanco, Dyson Mwandama, Memory Mkandawire, Elizabeth Kampira, Muluken Kaba, Alice Maida, Andrew F. Auld, Lindsay Kim, Reuben Mwenda, Howard Kress, James Kandulu, Thresa Sumani, Joseph Bitilinyu, Thokozani Kalua, Alash’le Abimiku

**Affiliations:** a Center for International Health, Education, and Biosecurity, Lilongwe, Malawi.; b Institute of Human Virology Nigeria, Abuja, Nigeria.; c Center for International Health, Education, and Biosecurity, University of Maryland School Medicine, Baltimore, MD, USA.; d Institute of Human Virology, University of Maryland School Medicine, Baltimore, MD, USA.; e University Research Co. Lilongwe, Malawi.; f Division of Global HIV and TB, U.S. Centers for Disease Control and Prevention, Lilongwe, Malawi.; g U.S. Public Health Service, Atlanta, GA, USA.; h Diagnostic Department, Ministry of Health, Lilongwe, Malawi.; i Department of Medicine, University of Maryland School Medicine, Baltimore, MD, USA.; j Division of Global Health Sciences, Department of Epidemiology and Public Health, University of Maryland School of Medicine, Baltimore, MD, USA.

## Abstract

**Introduction:**

As part of a laboratory strengthening program in Malawi to achieve and maintain International Organization for Standardization (ISO) 15189 accreditation, we intended to mentor selected HIV molecular laboratories to achieve this accreditation. Due to the COVID-19 pandemic, mentorship pivoted to a hybrid model using an Internet-based approach and on-site mentorships. We describe the implementation of this strategy, successes, and challenges.

**Methods:**

We conducted weekly, 1-hour virtual mentorship sessions for the 5 initial laboratories (cohort 1) selected based on their Stepwise Laboratory Quality Improvement Process Towards Accreditation (SLIPTA) performance score of 3 or more stars. Laboratories presented updates and supporting documents electronically, and trainings were conducted virtually. In September 2020, when travel restrictions were relaxed, we initiated hybrid mentorships and audits for cohort 1 laboratories. The same hybrid approach was used to mentor 4 additional laboratories in cohort 2. We performed descriptive analysis, and the Wilcoxon signed-rank test was used to compare the training pre-and post-test scores.

**Results:**

Between March 2020 and May 2023, the team completed a total of 54 virtual mentorship sessions and 20 on-site visits across 9 laboratories. Overall, the team conducted 8 training sessions for 35 laboratory quality officers. Median score improvement (pre-test vs. post-test scores) was observed across individual trainings and across cohorts (*P*<.01). At the end of cohort 1, 4 of 5 (80%) laboratories were accredited. One laboratory that did not reach accreditation joined cohort 2. At the end of the mentoring period, all 5 cohort 2 laboratories were accredited.

**Conclusions:**

We demonstrated that using a hybrid mentorship model for accreditation was a successful strategy during the COVID-19 pandemic. For the first time in Malawi, this strategy resulted in accrediting 9 of the 10 HIV molecular laboratories in 3 years at a reduced cost. Continuous mentorship is key in the maintenance of accreditation.

## Abstract (precis)

We describe a valuable hybrid mentorship and training model for supporting medical laboratories in low-resource settings in improving laboratory quality management systems and achieving accreditation.

## Teaser

**Key Findings:**

- Combining virtual and on-site mentorship sessions and training can be an effective strategy to support laboratories seeking accreditation.
- A reduced accreditation scope (number of test types) in the first stage of seeking accreditation can aid the accreditation process.
- Continuous mentorship is key in the maintenance of accreditation.

**Key Implication:**

- Hybrid mentorship can be a potential strategy for achieving and maintaining accreditation at a reduced cost.

## INTRODUCTION

Medical and diagnostic laboratories are crucial in managing patients in government and privately owned hospitals and clinics. These laboratories also support surveillance of disease outbreaks, as seen during the COVID-19 pandemic. Governments and donors have provided funding to sub-Saharan African laboratories to improve their ability to respond to outbreaks.^1^^,^^2^ To respond to disease outbreaks, laboratories must implement a laboratory quality management system (LQMS) to ensure that generated laboratory results are accurate, reliable, and timely.

To support laboratories in resource-limited settings in implementing an LQMS, the World Health Organization Regional Office for Africa, in collaboration with the U.S. Center for Disease Control and Prevention (CDC), developed the Stepwise Laboratory Quality Improvement Process Towards Accreditation (SLIPTA) program and launched a complementary training program called Strengthening Laboratory Management Toward Accreditation (SLMTA) in 2009.^3^^,^^4^ The SLMTA/SLIPTA is a stepwise improvement approach aimed at providing guidance and assistance to medical laboratories in achieving International Organization for Standardization (ISO) 15189, a standard that focuses on quality and technical competence to improve patient care and outcomes. SLIPTA scores laboratories using a checklist divided into 12 sections based on the LQMS essentials (Box).^4^ Over a thousand laboratories across 56 countries, mainly in Africa, Asia, Latin America, and the Caribbean, have implemented SLMTA. Since 2011, the Malawi Ministry of Health (MOH) and laboratory implementing partners have made significant efforts to establish and implement an LQMS in Malawi through the SLMTA/SLIPTA programs and ISO 15189 standards. However, no government laboratory had ever been accredited.

BOX World Health Organization Stepwise Laboratory Quality Improvement Process Towards Accreditation Checklist and Scoring

The World Health Organization Stepwise Laboratory Quality Improvement Process Towards Accreditation (SLIPTA) Checklist^5^ includes 334 questions with 275 points organized into the following 12 sections:

1. Documents and records
2. Management reviews
3. Organization and personnel
4. Client management and customer service
5. Equipment
6. Internal audit
7. Purchasing and inventory
8. Process control and internal and external quality assessment
9. Information management
10. Corrective action
11. Occurrence management and process improvement
12. Facilities and safety

Laboratories are scored during audits and recognized on a 0 to 5 ascending star scale:

- No stars (0–150 points); <55%
- 1 star: (151–177 points); 55%–64%
- 2 stars: (178–205 points); 65%–74%
- 3 stars: (206–232 points); 75%–84%
- 4 stars: (233–260 points); 85%–94%
- 5 stars: (261–275 points); ≥95%

The University of Maryland Baltimore (UMB) Center for International Health, Education, and Biosecurity (CIHEB) provided technical support to all molecular laboratories that support HIV viral load and early infant diagnosis (EID) testing in Malawi as part of the U.S. President’s Emergency Plan for AIDS Relief (PEPFAR) program. CIHEB supported Malawi through a CDC-funded laboratory strengthening program referred to as AMPLIFY (Accelerating Malawi’s PEPFAR Laboratory Logistics and Infrastructure for Quality) that provided laboratory reagents and supplies and technical support to implement the national HIV program. A key priority for the AMPLIFY project was to achieve and maintain ISO 15189 accreditation for all molecular laboratories in Malawi. In January 2020, UMB began mentoring 5 molecular laboratories that achieved a SLIPTA performance score of 3 or more stars. Amid planning for in-person mentorship visits with the laboratories in March 2020, the COVID-19 pandemic ensued, and the country faced lockdowns, limitations on congregating, and travel restrictions. Due to the pandemic, the social distancing measures required UMB to be innovative and incorporate web-based strategies for mentoring. We describe the implementation of UMB’s hybrid mentoring approach to build a robust LQMS and achieve ISO 15189:2012 accreditation in 9 molecular laboratories in Malawi. This is Malawi’s first experience accrediting molecular labs and using this hybrid mentoring approach.

We describe Malawi’s first experience accrediting HIV molecular labs and using this hybrid mentoring approach.

## METHODS

### Study Setting

From all 10 HIV molecular laboratories that support national HIV and TB programs across 27 districts in Malawi, in January 2020, the MOH selected 5 molecular laboratories to proceed to accreditation for the first cohort (Table 1). To qualify for mentorship to prepare for accreditation, each laboratory had to have been part of the SLMTA/SLIPTA program for a minimum of 3 years and had to have achieved a SLIPTA exit score of 3 or more stars. During the selection process, we considered the magnitude of the volume of testing, human resources, and facility/laboratory management support.

**TABLE 1.** Characteristics of Malawi HIV Molecular Laboratories Selected for Mentorship to Achieve ISO 15189 Accreditation

**#**	**Laboratory Type**	**Sample Type**	**Accreditation Scope**	**Testing Platform**	**Staff, No.**	**Distance From Lilongwe/ Central Lab, km**	**Star Recognition**
**Cohort 1**							
1	Public/NGO	DBS/plasma	HIV viral load	Abbott m2000	8	60 km	3
2	Public/NGO	DBS/plasma	HIV viral load	Abbott m2000	8	43 km	3
3	Reference	DBS	HIV viral load	Abbott m2000	14	0 km	4
4	Private/public	DBS/plasma	HIV viral load	Abbott m2000	12	20 km	3
5	District hospitala	DBS	HIV viral load, EID, TB, chemistry, serology, blood banking, and hematology	GeneXpert	8	0 km	3
**Cohort 2**							
1	Central hospital	DBS	HIV viral load	Abbott m2000	8	55 km	4
2	Central hospital	DBS	HIV viral load	Abbott m2000	8	46 km	1
3	Central hospital	DBS/plasma	HIV viral load	Abbott m2000	8	7 km	4
4	Central hospital	DBS/plasma	HIV viral load	Abbott m2000	8	10 km	3
5	District hospitala	DBS	HIV viral load, EID, TB	GeneXpert	8	0 km	4

Abbreviations: DBS, dried blood sample; EID, early infant diagnosis; NGO, nongovernmental organization; ISO, International Organization for Standardization; SLIPTA, Stepwise Laboratory Quality Improvement Process Towards Accreditation.

^a^ This laboratory was part of both cohort 1 and cohort 2, however, it reduced its scope for cohort 2.

In March 2021, a second cohort of 5 molecular laboratories was selected based on the same inclusion criteria, including 1 laboratory from cohort 1. One laboratory in the second cohort that scored 1 star during their SLIPTA exit audit was included, as it was critical to have 1 major laboratory accredited per region. Including this laboratory also allowed us to evaluate the feasibility of mentoring a 1-star laboratory to accreditation in 12 months.

The 9 selected laboratories included 6 government public health molecular laboratories and 3 private laboratories. The accreditation scopes for the laboratories were HIV viral load and EID; only 1 laboratory in cohort 1 had a broader testing scope.

### Mentorship Strategy

**FIGURE 1:**
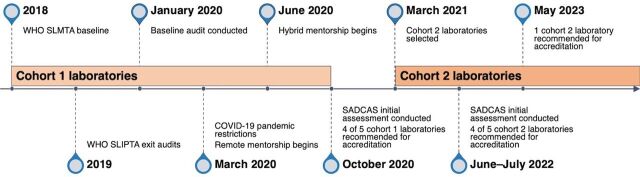
Mentorship and Accreditation Timelines for HIV Molecular Laboratories in Malawi Abbreviations: SADCAS, Southern African Development Community Accreditation Service; SLIPTA, Stepwise Laboratory Quality Improvement Process Towards Accreditation; SLMTA, Strengthening Laboratory Management Toward Accreditation; WHO, World Health Organization.

In January 2020, UMB began on-site mentorship of Cohort 1 laboratories. In March 2020, UMB implemented a remote mentorship model comprising (1) weekly virtual mentorship sessions, (2) virtual trainings, and (3) virtual internal audits (Figure 1).

#### Mentorship Sessions

The AMPLIFY project had a total of 5 mentors who mentored and prepared the 9 laboratories for ISO 15189 accreditation. For each of the laboratories selected for accreditation, UMB developed a mentorship schedule and tools to conduct an average of 4 1-hour virtual mentorship sessions per month (1 per week) via Zoom. Laboratories were supported with airtime, stationery, desktop computers, tablets, and printers to implement these sessions. Participation in the virtual mentorship calls was open to each laboratory’s manager and quality manager.

During the virtual sessions, laboratories presented updates on internal audits conducted that identified nonconformities classified as either major or minor based on their direct impact on patient care. A nonconformity was classified as major if it met any of the following criteria: (1) it had a direct impact on patient care or management, (2) it had an impact on QMS management or implementation, and (3) it was recurring in the system. A minor nonconformity was a general noncompliance to rules, regulations, or procedures that did not meet the previous criteria.

After each mentorship session, mentors followed up via phone calls and emails to support the laboratories through their LQMS implementation. After the COVID-19 restrictions were relaxed in June 2020, UMB supplemented the virtual mentorship sessions with monthly on-site 5-day mentorship visits at each laboratory.

After the COVID-19 restrictions were relaxed in June 2020, UMB supplemented the virtual mentorship sessions with monthly on-site 5-day mentorship visits at each laboratory.

Each laboratory underwent 1 year of mentorship, including 4 on-site audits, before its first assessment by the accrediting body Southern African Development Community Accreditation Service (SADCAS).

#### Virtual Training

UMB planned and conducted trainings virtually on Zoom for laboratory managers, technical personnel, and quality officers. Trainings were designed to cover gaps identified during internal audits and interactions with the laboratory quality officers. Training topics covered included how to conduct internal audits, quality risk management, and method verification to determine how suitable an analytical procedure was for its intended use and under the intended testing conditions. UMB mentors delivered these trainings using case studies for each training. Training effectiveness was mainly measured through the laboratory audits/assessments; however, pre-post assessments via Google Forms were performed in later trainings.

#### Internal Audits

To ensure laboratories’ readiness for the SADCAS assessments and check their Internet connectivity, in September 2020 (3 weeks before the SADCAS assessments), we initiated hybrid (virtual followed by on-site) audits in all 5 cohort 1 laboratories. UMB mentors and MOH staff conducted “dry-run” internal audits using the SADCAS checklists for different areas, including a management and technical requirements assessment, a vertical assessment, a witness of activity form, and a proficiency testing/calibration assessment. These checklists were linked to an ISO 15189:2012 clause for management and technical requirements.

The nonconformities were captured in Microsoft Excel sheets and shared with the laboratory quality managers for remediation. All 5 laboratories addressed and closed their nonconformities within the 25 working days usually allowed by SADCAS.

For cohort 2, we implemented the same hybrid audit and mentorship approach, tailoring individual and group mentorship to address specific gaps identified during laboratory audits.

### Southern African Development Community Accreditation Service Assessments

SADCAS, based in Botswana and registered in 2005, provides accreditation services to the Southern African Development Community region, which includes Malawi. All cohort 1 and 2 laboratories were enrolled under SADCAS for ISO 15189:2012 accreditation. After the document review process, SADCAS assigned 2 auditors per laboratory and scheduled the initial assessments. Cohort 1 initial assessments were conducted in October 2020, and cohort 2 initial assessments were conducted in June–July 2022.

Before the COVID-19 pandemic, SADCAS and other accrediting bodies conducted in-person assessments. During the COVID-19 travel restrictions and government policies, SADCAS implemented a fully remote assessment approach using Zoom video and instant messaging platforms to virtually review LQMS documents and processes in the laboratory.^5^ SADCAS resumed on-site assessments in September 2022.

### Data Collection and Analysis

We used the SADCAS checklists to conduct and document audits; however, we developed a Microsoft Excel template to capture and monitor nonconformities and corresponding timelines for each audit. The audits were classified into baseline, dry-run, and SADCAS audits. Training pre- and post-test scores were entered into a Microsoft Excel spreadsheet. Minutes were recorded at each meeting to keep track of participants and any connectivity issues. We performed descriptive analysis using medians, interquartile ranges (IQR), and proportions of the test scores. Additionally, pre- and post-test scores were compared using the Wilcoxon signed rank test, which allowed us to compare 2 dependent samples while accounting not only for the direction of the change but also its magnitude.^6^ The UMB team took advantage of its enterprise resource planning software used to process procurements and travel to extract cost data.

### Ethical Approval

The University of Maryland, Baltimore Institutional Review Board (HP-00094192) and National Health Science Research Committee (P 815/2020) approved the AMPLIFY protocol. This project was also reviewed in accordance with the CDC human research protection procedures. The Institutional Review Boards gave this protocol a “not human research” determination. Only aggregated and non-identifiable data were used for this analysis.

## RESULTS

### Mentorship Sessions Conducted

Figure 1 illustrates the mentorship strategy implemented in the 9 selected HIV molecular laboratories.

Between March 2020 and October 2020, we conducted a total of 28 weekly 1-hour virtual mentorship sessions for cohort 1 laboratories. Although each laboratory was given a monthly Internet bundle (US$19), attendance at the virtual meetings was impacted by connectivity issues (i.e., failure to connect to the call, call dropped-off, and audio challenges). Other issues, such as lack of power, high workload, or limited time to prepare for the call, may have also played a role in meeting attendance. The average annual lack of attendance due to connectivity issues was reduced from 18% in 2020 to 4% in 2023 as a result of UMB creating a WhatsApp group for meeting reminders, supporting the laboratory teams in selecting an Internet provider with the best connectivity for each laboratory location, and recommending joining meetings ahead of mentorship meetings to troubleshoot connectivity before the start of each meeting.

Additional on-site mentorship sessions that lasted 5 days were reintroduced to cohort 1 laboratories in June 2020, and a total of 10 on-site sessions were conducted for cohort 1. For cohort 2 laboratories, we conducted 26 virtual and 10 on-site mentorship sessions between March 2021 and May 2023. Within Lilongwe, on-site mentorship visits of 5 days cost US$38 per laboratory. Depending on the laboratory’s location outside of Lilongwe, on-site mentorship visits cost US$910–US$1156 per laboratory, considering fuel, per diem, and accommodations.

### Trainings Conducted

Across all trainings, there was a significant score improvement when comparing pre- and post-test scores.

**FIGURE 2:**
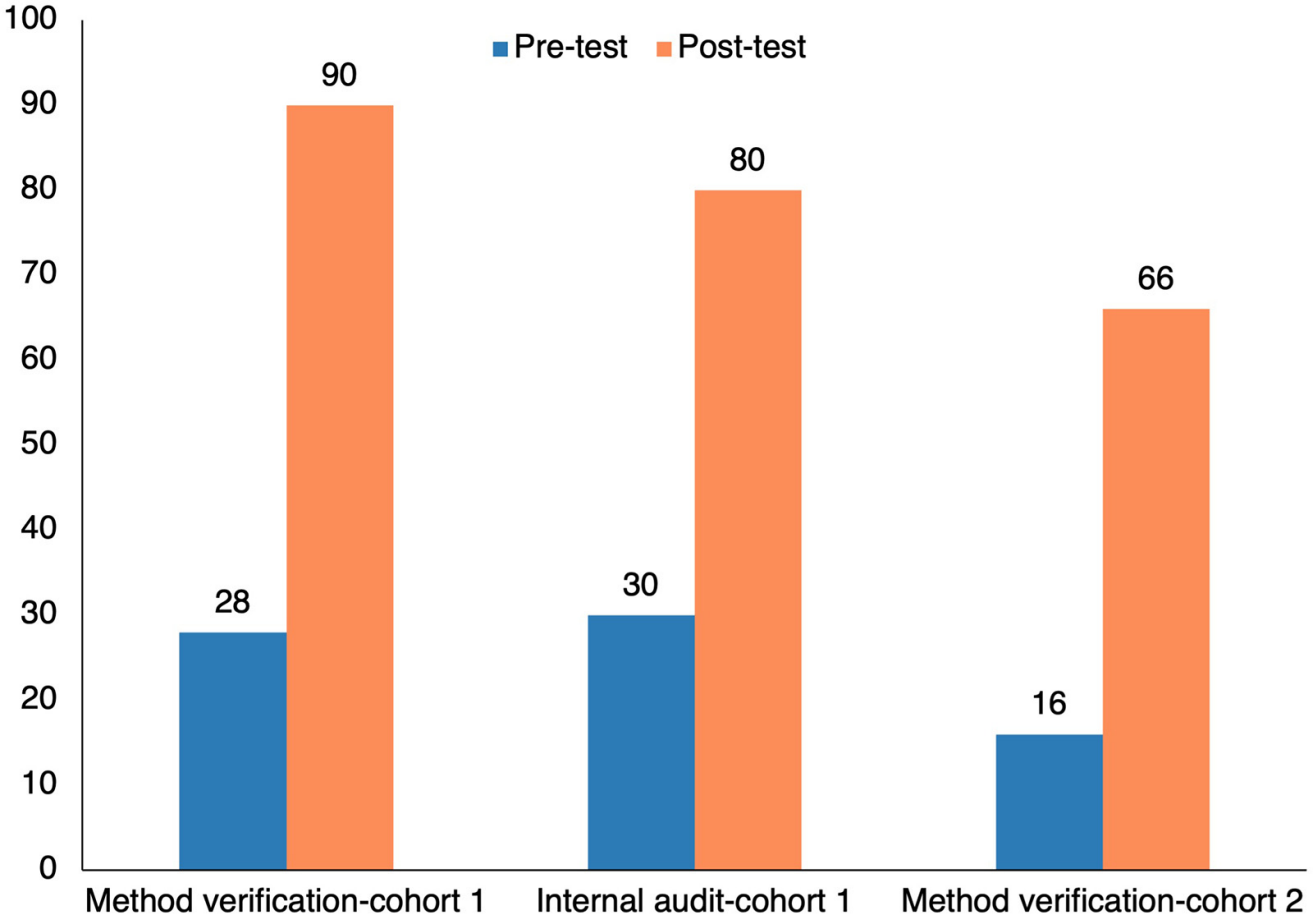
Median Score of Pre- and Post-Tests for HIV Molecular Laboratory Trainings, Malawi^a^ ^a^ All post-test scores represent a statistically significant difference (*P*<.01).

From January to October 2020, we organized a total of 6 trainings (3 virtual and 3 on-site) covering various topics related to the LQMS for 22 laboratory personnel in cohort 1 laboratories. For the pre-test assessment, the median score was 28 of 100 (IQR=20–50). The median post-test score was 90 of 100 (IQR=85–95). The median score improvement between the 2 tests was 55 (IQR=40–65, *P* value <.01) (Figure 2). The percentage of participants with a score above 70 increased from 5% to 95%. Additionally, we trained 25 quality officers and MOH mentors in cohort 1 on the internal audit process. The median pre-test score was 30 of 100 (IQR=20–40); the median post-test score was 80 (IQR=80–90). The median score improvement between the 2 tests was 50 (IQR=40–60, *P* value <.01) (Figure 2). The percentage of participants with a score above 70 increased from 0% to 81%.

Between March and April 2022, for cohort 2 laboratories, we conducted 2 additional trainings with 13 individuals (laboratory managers and quality officers) in quality risk management and method verification. The pre-test median score was 16 (IQR=16–21), and the median post-test score was 66 (IQR=50–80). The median score improvement between the 2 tests was 47 (IQR=26–63, *P* value <.01) (Figure 2). The percentage of participants with a score above 70 increased from 0% to 38%.

### Internal Audits Conducted and Nonconformities Found

For cohort 1, the baseline internal audit in January 2020 found a median of 35 nonconformities (IQR=30–35). When SADCAS conducted its initial assessment in October 2020, it reported a median of only 12 nonconformities (IQR=11–15). Between the 2 audits, there was a median reduction of 20 nonconformities (IQR=14–23, *P*=.06), indicating a decrease in the number of gaps and evidence of improvement in the laboratory staff performance and the functional quality system (Table 2).

**TABLE 2.** Nonconformities Identified During Baseline Audit and SADCAS Assessments of Malawi HIV Molecular Laboratories

	**Nonconformities, No.**									
**Laboratory**	**Baseline Audit**			**SADCAS Assessment**			**Change**			
	**Major**	**Minor**	**Total**	**Major**	**Minor**	**Total**	**Major**	**Minor**	**Total**	***P* Value**
Cohort 1										
1	30	5	35	7	4	11	23	1	24	.06
2	20	9	29	6	9	15	14	0	14	
3	25	10	35	5	7	12	20	3	23	
4	20	10	30	5	5	10	15	5	20	
5	28	8	36	30	1	31	-2	7	5	
Cohort 2										
1	68	10	78	8	3	11	60	7	67	.06
2	44	8	52	12	4	16	32	4	36	
3	49	4	53	12	1	13	37	3	40	
4	45	7	52	5	6	11	40	1	41	
5^a^	30	1	31	11	2	13	19	-1	18	

Abbreviation: SADCAS, Southern African Development Community Accreditation Service.

^a^ Cohort 1 lab 5 transitioned to cohort 2 lab 5; therefore, its SADCAS assessment from cohort 1 was used as its baseline audit for cohort 2.

Laboratories in both cohorts addressed the number of nonconformities identified in audits.

For cohort 2, the baseline audit in March 2021 found a median of 52 nonconformities (IQR=52–53). When SADCAS conducted their audit in June–July 2022, they found a median of 13 nonconformities (IQR=11–13), showing a median reduction of 40 nonconformities (IQR=36–41, *P*=.06) (Table 2). Nonconformities at the initial assessment were in management (84%) and technical (16%) clauses. Common nonconformities included gaps in nonconformity management (clause 4.9), management review follow-up (clause 4.15), internal audits (clause 4.14), customer surveys (clause 4.14.3), standard operating procedures (SOP), structure (clause 4.3) and training records for technical staff (clause 4.13, 5.15).

### Accreditation Results

Following the formal initial SADCAS assessments, 4 of 5 laboratories (80%) from cohort 1 and 5 of 5 laboratories (100%) from cohort 2 laboratories were recommended for ISO 15189:2012 accreditation in October 2020 and June 2022–May 2023, respectively (Table 3). The laboratory that did not achieve accreditation in cohort 1 in October 2020 was the only laboratory with a broad scope of testing types (HIV viral load, EID, TB, chemistry, serology, blood banking, and hematology) in cohort 1. This laboratory continued mentorship as part of cohort 2 with a narrower scope (HIV viral load, EID, TB) and achieved accreditation.

**TABLE 3.** Accreditation Status for Malawi HIV Molecular Laboratories in Mentorship Program

**Laboratory**	**Date Mentorship Began**	**Accreditation Status/Date**
**Cohort 1**		
1	January 2020	October 27, 2020
2	January 2020	October 16, 2020
3	January 2020	October 22, 2020
4	January 2020	October 29, 2020
5^a^	January 2020	Not successful
**Cohort 2**		
1	March 2021	June 8, 2022
2	March 2021	June 28, 2022
3	March 2021	May 25, 2023
4	March 2021	June 12, 2022
5^a^	March 2021	July 27, 2022

^a^ This laboratory was part of both cohort 1 and cohort 2, however, it reduced its scope for cohort 2.

Of the 9 accredited laboratories, 1 laboratory in cohort 1 had its accreditation temporarily suspended due to a delay in clearing the nonconformities identified at a 6-month post-accreditation surveillance SADCAS assessment. The laboratory cleared its nonconformities and was reinstated by SADCAS 8 months after its suspension.

## DISCUSSION

Conducting virtual laboratory mentorship at the peak of the COVID-19 pandemic was initially challenging but was effective in kick-starting the implementation of LQMS toward accreditation. We introduced hybrid mentorship as a new and innovative strategy to mentor and support laboratories as they established their LQMS and prepared for the SADCAS international accreditation. Interestingly, other programs have recorded varying successes with virtual mentorship.^7^^–^^9^

We find hybrid or virtual mentorship a more cost-effective strategy than on-site mentorship, as others have reported.^10^ Supporting 1 laboratory with Internet connectivity for a year costs US$228, and conducting a 5-day on-site mentoring visit each month for a year (12 trips a year total) would cost US$460 to US$14000, depending on the laboratory location (inside or outside Lilongwe). Moreover, the latter cost does not consider the extra time and coordination needed in addition to international travel for mentors from outside the country, especially for countries, such as Malawi, that are just embarking on the accreditation exercise. Fully traditional on-site mentorship visits lasted 2 weeks per site every month and would cost more.

Having laboratories move from the SLIPTA program to ISO 15189 accreditation requires intensive mentorship and alignment of documents. The goal of SLIPTA is to provide a stepwise approach for laboratories to achieve ISO accreditation after exiting the program. The MOH led the implementation of the SLIPTA program from inception, providing oversight to ensure LQMS implementation at district-level laboratories. The Diagnostic Department of the MOH implemented a 5-year laboratory strategic implementation policy plan and included the SLIPTA program as a key component for the country. All 9 laboratories that achieved ISO 15189 accreditation in our 2 cohorts had completed the SLIPTA program, which provided an approach to systematically implement an LQMS in preparation for ISO 15189 accreditation through a series of trainings and development of tools, similar to other laboratories on the African continent. Some of the challenges faced by laboratories in the preparation for ISO 15189 accreditation post-SLIPTA audit were being understaffed or requiring staff to multitask, specifically in government-owned facilities.^11^^,^^12^

During the remote assessment and the transition to a hybrid strategy, improvements were measured by the UMB mentors by the number of nonconformities found at specific audits and the ability of the laboratories to address these nonconformities within the specified timeline of 25 working days, aligning with SADCAS’s timeline.^7^^–^^9^ While on-site or in-person mentorship has the benefits of hands-on training, the implementation of virtual audits with videos to guide these assessments can be extremely efficient, allowing more laboratories to be assessed at once without the delay and expense of travel.^13^ This is especially effective during outbreaks and pandemics, as demonstrated during the COVID-19 pandemic. SADCAS also established remote assessments during the pandemic and, more recently, retransitioned to on-site assessment in 2022.

Overall, implementation improved with time, as the team was more experienced in mentoring laboratories toward international accreditation by the time cohort 2 started. Full participation in virtual calls is critical for a successful hybrid approach to mentorship. A highly committed and involved laboratory staff is essential. Facility management should nominate dedicated staff who can commit sufficient time to the process. Our team also learned that some activities can be carried out remotely, such as reviewing standard operating procedures, policies, and records, while others are better conducted on-site, such as assessing documentation and dry-run audits.

Despite the restrictions and challenges caused by the COVID-19 pandemic, our mentorship strategy resulted in the successful accreditation of 9 laboratories (90% of all HIV molecular laboratories in Malawi at the time) within 2 years. The 1 laboratory that did not achieve accreditation in cohort 1 had an extensive scope (HIV viral load, EID, TB, chemistry, serology, blood banking, and hematology) that added complexity to the preparation process due to the involvement of more staff members and the need to complete additional documentation, making the accreditation process more challenging. The laboratory required additional time to streamline the process and narrow the scope to only 2 recommended scopes (HIV and TB), allowing for a more focused approach.

Despite the restrictions and challenges caused by the COVID-19 pandemic, our mentorship strategy resulted in the successful accreditation of 9 HIV molecular laboratories in Malawi within 2 years.

Another laboratory faced the suspension of its accreditation due to a lack of adherence to the acquired standard, emphasizing the significance of continuous education, mentorship, and management support in maintaining accreditation, particularly in response to staff attrition.^13^ It is critical to ensure each laboratory embraces the processes of an accredited laboratory as the new standard, not just a 1-time achievement and then revert to old ways. In addition, the laboratory with an exit SLIPTA score of 1 star in cohort 2 needed additional mentorship and contact time (i.e., longer on-site visits, additional virtual calls with technical leads and advisors, and additional internal audits) compared to the other laboratories, as expected. However, this laboratory also achieved accreditation.

### Limitations

Our analysis was limited by the inability to directly compare the effectiveness of remote versus hybrid strategies, as the implementation was fully virtual only for a few months when COVID-19 pandemic restrictions were lifted. However, the program always intended to incorporate the advantages of the remote strategy with the traditional on-site strategy instead of fully transitioning to remote mentorship after the pandemic restrictions were lifted. Additionally, as this was Malawi’s first experience with ISO accreditation, we were unable to compare the effectiveness of the hybrid mentorship approach with a traditional on-site approach, as we lacked an appropriate comparison group. However, a descriptive snapshot of the implementation of this strategy highlighting its strengths and challenges is still informative to other countries in sub-Saharan Africa starting their ISO accreditation process.

Notably, there are limitations with a hybrid mentorship program, including poor Internet connectivity, especially at remotely located laboratories, and poor meeting attendance. The attendance improved over time. However, hybrid mentorship offers numerous advantages and is a valuable strategy for implementation in resource-limited settings such as Malawi. A hybrid LQMS mentorship approach that includes limited on-site visits and virtual mentorship can augment efforts toward preparing medical laboratories for international accreditation. The project’s emphasis on continuous improvement and virtual mentorship has helped promote lasting excellence in laboratory practices, benefiting patients and health care providers. The AMPLIFY project additionally built in-country capacity, increasing the pool of subject matter experts through the training of MOH mentors and assessors to augment mentorship efforts within the country. These master-trained MOH mentors and assessors participate in routine on-site mentorships, LQMS training, and internal or dry-run audits with UMB mentors. This was part of the sustainability strategy for ISO 15189 accreditation in Malawi beyond the AMPLIFY project. We did not report any data before the SLIPTA exit assessments and scores from the exit assessments, as CIHEB was not the implementing partner for this program.

## CONCLUSION

Overall, we describe a valuable model for supporting medical laboratories in achieving ISO 15189 accreditation, particularly in low-resource settings where continuous on-site mentorship may not be sustainable due to the limitation of subject matter experts in the country and the associated high costs for external mentors. Our model also provides an effective strategy to use during disease outbreaks and pandemics, when laboratories must continue to conduct essential tests to support national outbreak response and surveillance efforts. We have demonstrated that during pandemics, essential laboratory activities, such as training and mentorship, can be conducted using a hybrid approach. Although our findings indicate that hybrid mentorship was beneficial in preparing the laboratories for international accreditation, we noted that highly motivated staff, adequate administrative management support, and good Internet services played an important role in its success. Virtual mentorship and training can be combined with on-site mentorship in selected activities. Continuous mentorship is important for newly accredited laboratories to ensure retention of accreditation status.

## Notes

**Peer Reviewed**

**First Published Online:** November 15, 2024.
